# Addressing the disparities: the approach to the African American patient with multiple myeloma

**DOI:** 10.1038/s41408-023-00961-0

**Published:** 2023-12-18

**Authors:** Manisha Bhutani, Brandon J. Blue, Craig Cole, Ashraf Z. Badros, Saad Z. Usmani, Ajay K. Nooka, Leon Bernal-Mizrachi, Joseph Mikhael

**Affiliations:** 1 Department of Hematologic Oncology and Blood Disorders, Atrium Health Levine Cancer Institute/Wake Forest School of Medicine, Charlotte, NC USA; 2 Department of Malignant Hematology, H. Lee Moffitt Cancer Center and Research Institute, Tampa, FL USA; 3 Division of Hematology and Oncology, Michigan State University, College of Human Medicine/Karmanos Cancer Institute at McLaren Greater Lansing, Lansing, MI USA; 4 Department of Medicine, University of Maryland Marlene and Stewart Greenebaum Comprehensive Cancer Center, University of Maryland School of Medicine, Baltimore, MD USA; 5 Multiple Myeloma Service, Department of Medicine, Memorial Sloan Kettering Cancer Center, New York, NY USA; 6 Department of Hematology and Medical Oncology, Winship Cancer Institute, Emory University School of Medicine, Atlanta, GA USA; 7 Translational Genomics Research Institute, City of Hope Cancer Center, Phoenix, AZ USA; 8 International Myeloma Foundation, Studio City, CA USA

**Keywords:** Health care, Myeloma

## Abstract

There are significant disparities with regards to incidence, timely diagnosis, access to treatment, clinical trial participation and health care utilization that negatively impact outcomes for African American patients with multiple myeloma. Health care providers have a role in ameliorating these disparities with thoughtful consideration of historical, sociocultural, individual and disease characteristics that influence the care provided to African American patient population. This review by a group of experts committed to health disparity in multiple myeloma provides a snapshot of disparities at both biologic and non-biologic levels, barriers to clinical care, and best practices to ensure that African American patients receive the best care available.

## Introduction

There are many reported associations between race and ethnicity and multiple myeloma incidence and outcomes. The multiple myeloma disparities among African American patients are complex and multifactorial. The contributing factors are broadly divided into two major categories based on whether they are related to disease biology (biologic) or to social determinants (non-biologic). Understanding the disparities and their underlying driving factors is important for practicing physicians as studies have shown that providers are less likely to deliver effective treatments to African American patients when compared to their White counterparts, even after controlling for characteristics like class, health behaviors, comorbidities, and access to health insurance and health care services [1–3]. We provide a snapshot of disparities at both biologic and non-biologic levels, address clinical barriers to, and suggest best practices that can be adapted to produce equitable outcomes for African American patients diagnosed with multiple myeloma. It is important to recognize that the U.S. Census defined racial and ethnic groups selected by individual respondents reflect a social, geographic, and cultural definition of race recognized in this country. These population descriptors are unreliable proxies for biologic, anthropologic or genetic differences of patients’ racial and ethnic backgrounds [4]. In the absence of categories that are more granular and specific, the race and ethnicity, though not a biologic concept, can be considered a starting point from which to generate hypotheses about environmental exposures and social processes that produce disparities in health outcomes. African American and Black racial identities are not always interchangeable. While the term African American is a socially and politically meaningful identity for many people of African descent, some people prefer the term Black because they do not identify themselves as African [5]. However, for inclusivity and consistency, the racial term African American has been used throughout this article except when referring to the studies or population statistics where the preferred usage was the term Black to define the race. The two terms are not used interchangeably in this article unless both terms were formally used in the referenced study.

## Multiple myeloma facts and figures for African American population

Multiple myeloma is the number one hematologic malignancy among African American patients, with an estimated 7810 new cases and 2530 myeloma deaths in this population in 2022 [6]. While African American individuals currently represent 13.6% of the US population, they comprise roughly 20% (one in five) of the newly diagnosed multiple myeloma population [6]. Compared with White population, African American people have a two-fold higher incidence of multiple myeloma, and its precursor condition monoclonal gammopathy of undermined significance (MGUS) [7]. Reports suggest that excess risk of multiple myeloma in African American patients is due to increase in the risk of MGUS rather than an increase in the risk of progression from MGUS to multiple myeloma [8]. The lifetime probability of being diagnosed with multiple myeloma among African American men and women is 1.4 and 1.2, respectively, compared with 0.8 and 0.6, among White men and women, respectively [9]. The disparity becomes more pronounced among patients younger than 50 years where rates of multiple myeloma are 2.6 times higher in Black men and 3.3 times higher in Black women than the rates for White men and women, respectively [6]. From 2009 to 2018, incidence continued to increase steadily in Black women by ~2% per year, whereas the rate in Black men appears to be approaching stabilization [6]. Like incidence rates, multiple myeloma mortality rates are twice higher in African American patients than White patients. Estimates from recent US Surveillance, Epidemiology, and End Results (SEER) database indicate that the 5-year age-adjusted mortality rate (from 2016 to 2020) per 100, 000 persons is 7.3 in Black men vs. 3.7 in White men and 5 in Black women vs. 2.2 in White women (Myeloma — Cancer Stat Facts).

Therapeutic innovations over the past two decades have positively impacted the life expectancy of patients with multiple myeloma in general. The 5-year relative survival rate for multiple myeloma improved from 29% during 1975 through 1977 to 58% during 2011 through 2017 among Black patients versus 24% to 55%, respectively, among White patients [6]. SEER registry-based analyses and other studies, mostly from the era where treatment approaches combining proteasome inhibitors (PIs) and immunomodulatory drugs (IMiDs) were not so popular, have shown equal or superior disease-specific and/or overall survival for African American than non-Hispanic White patients after adjusting for demographic factors, comorbidities and/or treatment [2, 7, 10–13]. On the other hand, data from the Multiple Myeloma Research Foundation CoMMpass study showed an inferior survival for African American patients compared with White patients when a lower use of frontline triplet induction therapy was reported for African American cohort [14]. A large Veterans Affairs study with equal access to healthcare showed potentially superior survival for African American patients <65 years old compared with White patients with multiple myeloma [15]. Another real-world retrospective analysis comparing outcomes using Flatiron database from >200 cancer clinics in the US showed unadjusted median overall survival indexed to first line of therapy was 64.6 months for African American patients and 54.5 months for White patients [16]. Nonetheless, African American individuals have not experienced similar survival benefits from recent treatment advancements because of poor access to care, including delays in treatment, and underutilization of new effective treatments in both real-world settings and clinical trials [2, 10, 13, 17, 18]. The observed favorable survival of African American population reinforces the importance of extending equal access opportunities to all races diagnosed with multiple myeloma and understanding the biologic differences that underlie the racial disparities.

## Biologic disparities unique to African American population

i. *Hereditary and familial susceptibility*
  Having a family history of multiple myeloma or a related plasma cell dyscrasia is a strong risk factor for developing multiple myeloma and MGUS. First-degree relatives of patients with multiple myeloma have a 2–3 times higher risk of developing the disease [19, 20]. Case clusters of MGUS and multiple myeloma within families have also been reported. In a large, pooled data set investigating risk of multiple myeloma in context of family history, the association was particularly strong among African American individuals (odds ratio = 5.52, 95% CI: 1.87–16.27) [21]. Other studies with smaller sample size have also suggested that familial aggregation of multiple myeloma is stronger among African American individuals than European American individuals [22, 23]. These data suggest that genetic inheritance may play a role in increased incidence of multiple myeloma and its precursor condition in African American population. Further support to this hypothesis comes from higher prevalence of autosomal dominantly inherited hyperphosphorylated form of the paratarg-7 protein (pP-7) carriers among patients with multiple myeloma of African American descent compared to other ethnic groups [24]. Additionally, a meta-analysis of genome wide association studies (GWAS) has identified loci in African American populations potentially associated with multiple myeloma that are distinct from the risk alleles identified in European American populations [25]. As most GWAS populations in multiple myeloma studies have been European, there is a need to expand the diversity to elucidate the underpinnings of disease susceptibility and clinical differences observed between populations.
ii. *Obesity and related risk factors*
  Obesity is one of the established risk factors for both multiple myeloma and MGUS [26–28]. More than 60% of the adult US population falls in the overweight (BMI 25–29) and obese (BMI > 30) categories [29]. Among African-American adults, nearly 48% are clinically obese compared to 34.5% of non-Hispanic White individuals [30]. African American individuals also have a higher prevalence of other chronic risk factors, such as diabetes mellitus, hypertension, metabolic syndrome, dyslipidemia, insulin resistance and cardiovascular diseases, that are intimately associated with obesity [31, 32]. Obesity and other related conditions potentially activate molecular pathways that favor pathogenesis of MGUS and multiple myeloma. Since obesity is a modifiable risk factor, it is important to increase awareness of multiple myeloma risk among African American individuals with obesity.
iii. *Different disease biology*
  Distinct biological subtypes with a range of molecular and genetic features have been associated with disparate survival outcomes. One explanation for better prognosis of multiple myeloma among African American patients is that they have more indolent disease biology. Lower incidence of high-risk genomic profile, including *t*(4;14), *t*(14;16), *t*(14;20), and deletion of 17p, has been noted among African American patients compared with White patients [33–35]. Conversely, translocation *t*(11;14), a favorable prognostic cytogenetic feature, was found in 6 of 21 African American patients compared with none of 47 White patients (29% vs. 0%; *p* = 0.001) [36]. Although most studies have relied on self-reported race, a recent study that quantitatively measured African ancestry demonstrated the probability of having one of three specific subtypes, namely *t*(11;14), t(14;16), or *t*(14;20), was significantly higher in the 120 individuals with the greatest African ancestry (≥80%) compared with the 235 individuals with the lowest African ancestry (<0.1%) [37]. The presence of t(11;14) was associated with superior overall survival post autologous stem cell transplantation (ASCT) among African American patients compared with white patients, although co-occurrence of high-risk cytogenetic abnormalities [defined as del(17p), *t*(4;14), *t*(14;16), +1q21, and del(1p)] was observed more often in White patients (27%) than African American patients (21%), thus potentially confounding the results [38]. These observations highlight the differences in molecular events surrounding multiple myeloma pathogenesis and progression in African American and White patients.
iv. *Clinical characteristics*
  Race-specific difference in clinical characteristics has been reported in MGUS and multiple myeloma. African American individuals with MGUS have lower levels of monoclonal protein, an earlier age of onset, lower prevalence of IgM MGUS, and a higher frequency of abnormal serum free light chain (sFLC) ratios compared with White individuals [39–41]. Consequently, per the Mayo Clinic risk stratification model [42] (derived largely from White populations) that incorporates three adverse risk factors including an abnormal sFLC ratio, non-IgG MGUS, and M protein >1.5 g/dL, high-intermediate or high-risk MGUS is seen in comparatively low number of African American patients [41]. Like MGUS, African American individuals are more likely to develop multiple myeloma at a younger age and have adverse disease characteristics compared with white counterparts [7]. Median age of onset of multiple myeloma is about 4–5 years lower for African American patients compared with White patients. The proportion of African American patients diagnosed under 60 years of age was 35.3% vs. 16.5% for non-Hispanic White patients [43]. Younger age at diagnosis may portend better outcome, yet in clinical trials data, the median age of African American patients was older than that of the White patients (62 years compared to 58, respectively), implying that clinical trials are not capturing a representative Black patient population [44]. African American patients are more likely to have renal dysfunction and anemia at the time of diagnosis than non-Hispanic White patients [43, 45]. In pooled data from 9 large national cooperative group clinical trials, African American patients diagnosed with multiple myeloma had hemoglobin ≤10 g/dL and high mean LDH [12]. African American patients have the highest rate for all myeloma defining events, except bone fractures, which were high in White patients [46]. Compared to non-Hispanic White patients, non-Hispanic Black patients have higher incidence rates of solitary plasmacytomas and extramedullary plasmacytomas [47].

## Non-biologic disparities unique to African American population

i. *Systemic Racism*
  It cannot be underestimated how years of systemic racism has been a driver of health disparities in general [48–50]. Even independent of the economic factors described below, adverse health outcomes have been demonstrated because of systemic pressures that have marginalized the African American population [51]. This has resulted in less trust in the healthcare system, a greater proportion of uninsured individuals and reduced representation in the healthcare workforce.
ii. *Socioeconomic and Lifestyle disparities*
  Although a small fraction of the Black-White disparity can be attributed to biologic differences, most of it occurs in the context of broader inequality at social, economic, and structural levels. African American individuals have lower median incomes, are more likely to be unemployed, work low paying jobs, and often earn less for the same level of expertise, relative to their White counterparts [52]. Socioeconomic status is intricately linked to lifestyle and environmental risk factors. For example, African American individuals in the US are more likely to live in low-income areas that are exposed to higher levels of environmental pollution and psychosocial stressors [53]. Furthermore, socioeconomic status has implications on education, income, and health insurance. Financial distress associated with management of multiple myeloma from diagnosis through multiple relapses is enormous. The costs of therapeutic strategies such as ASCT, novel therapeutics, and cellular therapies and their impact on employment and disability disproportionately burden patients who are socioeconomically disadvantaged, many of whom are African American individuals.
iii. *Delay in Diagnosis*
  A delay in diagnosis is common in multiple myeloma for all patients—indeed, most patients will visit their primary care provider three times with signs and symptoms consistent with myeloma before the diagnosis of multiple myeloma is considered, as shown in a study from National Health Service in the UK [54]. Furthermore, a notable delay from disease diagnosis to receipt of treatment has been noted for African American patients. For example, in one study the average length of time between multiple myeloma diagnosis and start of treatment with a novel therapy was 5.2 months for African American patients compared to 2.7 months for White patients [10]. There are many potential reasons for the diagnostic and treatment delays including reduced access to primary care, mistrust in the healthcare system, financial barriers, poor physician-patient communication, and physician’s bias. Visits to a health care provider are sometimes not sufficient to ensure timely use of diagnostic and treatment services. Pain, which is one of the common presenting symptoms for multiple myeloma, is often misinterpreted and not properly recognized by physicians. Bone pain or low back pain are often misinterpreted as arthritis or osteoporosis related and not taken seriously. Although African American individuals have a biological predisposition for lower pain tolerance, it has been widely documented that African Americans are more at risk than Whites to receive poorer quality of care and under-treatment of pain [55]. Additionally, Black patients have also been shown to be less likely than White patients to undergo a complete initial diagnostic evaluation needed to complete staging, and proper imaging to test extramedullary disease [56]. Symptoms of multiple myeloma are often attributed to confounding diagnoses like anemia, diabetes, and chronic kidney disease that are more common in African American patients [57].
iv. *Disparities in access to quality care and clinical trials*
  Improving survival in multiple myeloma has been driven by many factors that include the use of combination therapy (Triplets), autologous stem cell transplants (Transplants), clinical trials (Trials), and CAR T cell therapy (T Cell therapy). All four “T”s have been less accessible to African American patients. Studies suggest differences in evidence-based treatment utilization, generally showing that African American patients are less likely to receive novel therapies like PI, IMiDs, ASCT, and targeted antibody therapy, and more likely to undergo ASCT later in their disease course [10, 44, 58, 59].

The use of CAR T cell therapy and bispecific antibodies in multiple myeloma is expanding for triple-class (PI, IMiDs, and anti CD38 antibody) exposed or refractory disease. Two BCMA-directed CAR T products, Idecabtagene vicleucel and Ciltacabtagene autoleucel, two BCMA-directed bispecific antibodies, Teclistamab and Elranatamb, and one GPRC5D bispecific antibody, Talquetamab were recently approved by the US FDA for use in patients with relapsed refractory multiple myeloma. Low enrollment for African American patients was reported in registrational clinical trials of idecabatgene vicleucel (6%) [60], ciltacabtagene autoleucel (18%) [61], Teclistamab (12.7%) [62], Elranatamb (7.3%) [63], and Talquetamab (11.4%) [64]. Only 35.9% of African American individuals lived in a county with a CAR T open trial, and of the ten states with the highest proportion of African American residents, six states (60%) had no (three states) or less than three clinical trial opened (three states) for either CAR-T or bispecific antibody [17].

Despite constituting 17.5% of patients in the real-world data, African American patients accounted for only 1.3% of clinical trial participants [44]. Among 2896 patients with newly diagnosed multiple myeloma who were enrolled in nine cooperative clinical trials over more than two decades, only 18% were non-White. The enrollment of African American US participants in pivotal clinical trials of multiple myeloma submitted to the FDA between 2003 and 2017 was disappointingly low at 4.5% [65]. In an extended pooled analysis between 2006 and 2019, African American patients still comprised 4% of the total population [18]. Eighteen of the 19 trials included in the analysis were global trials, with US patients representing 17% of the total population in these trials. Although African American patients were enrolled primarily in the US, in terms of absolute numbers, African American patients comprised ≤6% of the population enrolled in the US.

African American patients are also underrepresented in genomic sequencing databases and specimen acquisition studies. The inadequate representation of minority groups in research reduces our ability to generalize findings and create hypotheses because the unique biology of host and tumors from this subpopulation is not accounted for. Disparities are perpetuated as race-based differences in drug metabolism, toxicities and response rates are not considered in the development of innovative medical treatments [12].

## Addressing barriers to clinical care

Barriers in clinical care that African American patients face can be divided into two major overlapping categories that interact with each other. Patient-level barriers include systemic distrust in the medical and research system, lack of disease awareness, perceived risk of harm, lack of transportation, lack of access to treatment and clinical trials, lack of time, financial burden, fear of clinical procedures, and family issues. Treating physician–related barriers include implicit and explicit biases, cultural differences, lack of time, unwillingness to educate patients, referral bias, and fear that clinical trials create a financial or administrative burden to the practice. In the following sections, we provide high impact actionable solutions for some important barriers (Table 1).

**Table 1:** Barriers and recommendations for treatment of Blacks patients with multiple myeloma.

Barrier	Challenges	Solutions
Trust	Fear of unintended outcomes Fear of mistreatment	• Address concerns directly and openly • Engage patients in decision making
	Historical Stigma	• Strengthen the bond between doctor/patient
Awareness	Limited information or lack of information Misinformation on disease characteristics/risk Language fluency Written literacy deficiency Technology Access	• Community engaged education programs which involve caregivers and community members • Culturally appropriate education Tools • Transparent communication using culturally appropriate language • Allow informed consent to be available in native language • Improve the pipeline from primary care physicians to specialists to prevent delays in care
Clinical Trial Opportunities	Implicit and Explicit Bias regarding research	• Engage communities in clinical research to recruit underserved populations • Strategically open clinical trials near dense populated minority communities • Expand Eligibility Criteria • Decentralize clinical trials via telemedicine and remote data collection to involve community oncology practices
Access to Quality Care	Lifestyle/Behavioral Challenges	• Support medical transportation and healthcare rideshare programs • Develop innovative ways to offset medical costs(travel, lodging, medications) • Promote use of local laboratory/imaging testing facilities • Strengthen telemedicine visits

i. *Building Trust*
  Mistrust in the health care system may influence a patient’s experience in seeking medical care, accepting recommended treatments, and adhering to prescribed regimens. Multiple studies have shown that African American patients report lower levels of trust in health care providers than White patients. The mistrust is often rooted in past legacies of mistreatment and contemporary experiences of perceived racism and discrimination within patient-provider interactions. African American respondents report negative experiences with health care professionals, for example, the doctor assumed something about them without asking, talked down to them or did not treat them with respect, or did not believe they were telling the truth [66]. In a study that examined attitudes towards clinical trials among African American patients with multiple myeloma, the common reasons for not choosing to participate in a trial were fear of side effects, fear of receiving a placebo, and discomfort with being randomly assigned to a treatment [67]. Participants reported a significant level of distrust in medical research and doctors, saying that it was “very or somewhat likely” that doctors provide treatment as part of an experiment without patient consent [67]. In another study, African American patients were less likely to feel engaged in their treatment plan when compared to White patients [68].
  Clinicians and patient-facing staff have a key role in relation building by providing positive interactions. When interacting with all patients it is important to communicate clearly, consider their social context, and incorporate their perspectives and insights into the treatment decision process. Active and meaningful patient engagement means listening genuinely to their symptoms and complaints and spending time explaining them treatment and clinical trials. This is often described as culturally sensitive care, or the practice of cultural humility [69].
ii. *Improving disease Awareness*
  The lack of disease awareness among patients can lead to delayed diagnoses, which can negatively impact clinical courses and treatment outcomes. In a survey of more than 700 patients with multiple myeloma, 83% reported having no prior knowledge of multiple myeloma before their diagnosis [70]. Systemic factors such as limited educational opportunities and a lack of culturally tailored health information and services are health literacy barriers for African American population. Without proper awareness, those who are at the highest risk cannot take the necessary precautions to diagnose and treat the disease. Physicians should adopt culturally appropriate and interactive approaches and graphics to improve literacy related to multiple myeloma in African American patients in clinics and communities.
iii. *Improving time to diagnosis*
  Approximately two thirds of patients with multiple myeloma arrive at their presumed diagnosis through primary care [71]. Increased education of primary care providers is needed to facilitate both the suspicion of the diagnosis and the confirmation of the diagnosis through the accurate ordering of lab tests including the free light chain assay [72].
iv. *Cultivating Cultural competence*
  A culturally competent health care system provides a potential way to improve health outcomes and quality of care among patients. Cultural competence techniques include interventions such as racially or linguistically concordant clinicians and staff, culturally competent education and training, and culturally competent health education. Provider-patient racial concordance has been shown to improve patient outcomes and patient acceptance of longitudinal care [73]. Black physicians are vastly under-represented in oncology specialties, research careers and on medical school faculty. According to Association of American Medical Colleges, just 3% of medical oncologists are Black and/or African American [74]. Health care systems need to invest in recruiting, training, and integrating more racially concordant providers to reduce health inequities. As an example of this approach, the International Myeloma Foundation, in partnership with the National Medical Association has created a program for minority medical students to gain greater expertise in multiple myeloma and in health disparities. The students are matched to myeloma experts with further expertise in health equity. Each mentor-mentee pair is tasked to conduct a project in health disparities in multiple myeloma that is presented at the Annual Meeting of the National Medical Association. The program is called Medical Student Scholars for Health Equity in Myeloma and was piloted in 2023 (www.myeloma.org/medical-student-scholars-health-equity-myeloma).
  Cultural competence is required of all oncology professionals to build knowledge, skills, and attitudes for effective cross-cultural quality care for patients. African American culture has some common practices and values, including sharing, caring, maintaining family and community together, and praying for others. It is important for physicians to appreciate differences in health care values, and foster attitudes of humility, empathy, curiosity, respect, sensitivity, and awareness. Culturally congruent care happens when the needs, preferences, and expectations of patients, families, and communities are aligned with clinician knowledge, attitudes, and skills.
v. *Promoting Effective Communication*
  Keeping an open communication is vital to developing and maintaining mutual trust. Frequently patients are not fully prepared to discuss the possible treatment options effectively with their provider. Older adults in African American communities may trust in prayer, spiritual healers, and advice from family and friends. Instead of trying to dispel patients’ beliefs, providers should listen and validate their concerns. The providers can ask their patients in a nonjudgmental manner if they have any fears or concerns about their diagnosis and treatment and how do they feel their care is going. Providers must communicate verbally and non-verbally in a respectful and culturally appropriate way. Studies show that effective communication by oncology professionals is important to improve patient satisfaction [75]. Unfortunately, evidence consistently documents poor communication and less satisfaction with decision making for African American patients and their families [76, 77]. In focused group interviews, African American participants stressed that health care providers needed to know the person and family to tailor communication and emphasized that patients should be given sense of control for treatment choices [78]. African American patients place equal importance on patient-physician relationships that are characterized as emotionally supportive, include shared decision-making, and value the whole person. By focusing on improving communication, increasing transparency, and creating welcoming environments, physicians can engage patients more meaningfully.
vi. *Handling Implicit and Explicit bias*
  Racial bias is one of the factors that contributes to disparities in health and health care. Explicit racial biases are deliberative attitudes and beliefs that we operate at the conscious level and express through physical and verbal interaction or through more subtle means such as exclusion. Implicit bias, by contrast, operates at our unconscious level of awareness and can be in direct contradiction to our espoused beliefs and values. Physicians exhibit low levels of explicit bias, but implicit racial bias is automatically activated. One common implicit bias is that African American patients will be less compliant with a complex treatment regimen or clinical trial. The physicians’ association of this negative stereotype with African American patients potentially leads to differential patient referral to clinical trials thereby limiting opportunities among these racial and ethnic subpopulations [79]. It has been shown that the African American patients perceived communication as less patient-centered and supportive among oncologists who had higher levels of implicit bias [80]. A review of studies found that health care providers’ implicit racial bias with African American patients was associated with negative ratings of their clinical interactions, less patient confidence in recommended treatments, undertreatment of pain, poor provider communication, and views of African American patients as less medically adherent than White patients, and other ill effects [81].
  Pain management is an area where substantial racial biases in treatment of African American individuals’ pain have been well-documented [82]. This disparity has been attributed to factors from the provider level, system level, and patient level [83]. Physician-level bias has been associated with false beliefs that African American patients have greater pain tolerance, thicker skin, and feel less pain than White patients [84]. At patient-level, it has been noted that despite intense pain, older African American adults are hesitant to report or openly talk about pain. As providers we need to be aware of the unique barriers that African American patients experience; such barriers include but are not limited to: dislike of pills, fear of addiction, and bothersome side effects [85]. Education to dispel misinformation about race and pain and to explore implicit bias and ways to manage its harms should be part of health system–wide efforts.
vii. *Patient Navigation*
  Trained Navigators can help patients effectively navigate through cultural barriers impeding care and help them with referrals, identify primary care physicians, provide trusted information for shared decision-making. Navigators may be lay navigators (individuals from the community, with no clinical expertise, who relate to patients in a culturally appropriate manner and connect the community with the healthcare system), clinical navigators (nurses or social workers), or members of a multidisciplinary team of navigators who address a broad range of social and clinical needs.
viii. *Community engagement*
  Identification and collaboration with trusted communities (churches, hair salons, barber shops, fraternities/sororities, community centers, etc.) is critical to build and trust and credibility for both health systems and individual providers. A greater inclusion of community members on patient advocacy committees and clinical trial steering committees is also a valuable means to deepen community engagement. The International Myeloma Foundation has been a national and local platform of community engagement in the form of the M-Power program (M-Power – An International Myeloma Foundation Initiative). It is rooted in collaborating with both medical and non-medical groups to engage the community to raise awareness of multiple myeloma, its presenting symptoms, its impact in the African American community and means to overcome its health disparity.
ix. *Improving access to clinical trials*
  The National Institutes of Health (NIH) Revitalization Act of 1993 was established to enhance enrollment and retention of African American participants in NIH supported grant applications. Several strategies have been since implemented to increase recruitment rate of African American participants, including selection of sites that have diverse patient population, provision of additional funds, the 2010 launch of the FDA Office of Minority Health and Health Equity, establishment of FDA Drug Trials Snapshots, and the NIH mandate for race/ethnicity subgroup analysis in phase 3 trials. The NIH guidelines have contributed to an increase in minority inclusion. Large studies conducted in the US such as GRIFFIN [86] and DETERMINATION [87] are publishing a separate analysis and reporting on participant rates for African American population. Continued efforts are required to encourage enrollment on future studies to decrease the gap of health disparities. Eligibility criteria for trials should be as broad as possible so that they will better reflect the patients treated with those drugs in the real world. Physicians play a key role in encouraging African American patients to enroll in clinical trials and research. Increasing community involvement, screening African American individuals in geographically focused areas, incentivizing collaboration among diverse groups, and involving community hospitals in the recruitment to clinical trials will increase access for minority groups and result in a quicker enrollment process.

## Concluding remarks

The evolving understanding of the impact of racial diversity on clinical outcomes in multiple myeloma calls for the need for more racially and socioeconomically inclusive approaches to diagnosis and treatment. There are multiple points along the journey of patient with multiple myeloma that can be leveraged to improve patient care and reduce disparities. Understanding biological vulnerabilities at racial level is important when investigating cancer risks across races. Reducing disparities is a dynamic process that evolves over time. Addressing the presence of bias and discrimination directly and at the level of the individual is the key to improving cultural sensitivity and competence. Empowering individuals to report symptoms accurately, encouraging physicians to examine their own cultural beliefs and stereotypical perceptions, and modifying counterproductive beliefs and attitudes regarding care are some of the key best practices. Engaging community partners to raise awareness about clinical trials is a powerful tool to increase diversity. Ensuring that new innovations and treatment strategies are delivered equitably will engender a culture where quality is valued for all patients.
